# Indoleamine 2,3-dioxygenase is a novel prognostic indicator for endometrial cancer

**DOI:** 10.1038/sj.bjc.6603477

**Published:** 2006-11-21

**Authors:** K Ino, N Yoshida, H Kajiyama, K Shibata, E Yamamoto, K Kidokoro, N Takahashi, M Terauchi, A Nawa, S Nomura, T Nagasaka, O Takikawa, F Kikkawa

**Affiliations:** 1 Department of Obstetrics and Gynecology, Nagoya University Graduate School of Medicine, 65 Tsurumai-cho, Showa-ku, Nagoya 466-8550, Japan; 2 Division of Pathology/Clinical Laboratory, Nagoya University Graduate School of Medicine, Nagoya, Japan; 3 National Institute for Longevity Sciences, National Center for Geriatrics and Gerontology, Obu, Japan

**Keywords:** indoleamine 2,3-dioxygenase (IDO), endometrial cancer, prognostic factor, progression-free survival (PFS)

## Abstract

Indoleamine 2,3-dioxygenase (IDO) is a tryptophan-catabolising enzyme inducing immune tolerance. The present study aimed to investigate IDO expression and its prognostic significance in endometrial cancer. Indoleamine 2,3-dioxygenase expression in endometrial cancer tissues (*n*=80) was immunohistochemically scored as four groups (IDO−, 1+, 2+, and 3+). The high IDO expression (IDO2+ or 3+) in tumour cells was found in 37 (46.3%) of the 80 cases, and was positively correlated with surgical stage, myometrial invasion, lymph-vascular space involvement, and lymph node metastasis, but not with the histological grade. Patients with high IDO expression had significantly impaired overall survival and progression-free survival (PFS) (*P*=0.002 and *P*=0.001, respectively) compared to patients with no or weak expression of IDO (IDO− or 1+). The 5-year PFS for IDO−/1+, 2+, and 3+ were 97.7, 72.9, and 36.4%, respectively. Even in patients with early-stage disease (International Federation of Gynecology and Obstetrics I/II, *n*=64), the PFS for IDO2+/3+ was significantly poor (*P*=0.001) compared to that for IDO−/1+. On multivariate analysis, IDO expression was an independent prognostic factor for PFS (*P*=0.020). These results indicated that the high IDO expression was involved in the progression of endometrial cancer and correlated with the impaired clinical outcome, suggesting that IDO is a novel and reliable prognostic indicator for endometrial cancer.

Endometrial cancer is the most common malignancy of the female genital tract (Jemal *et al*, 2004). In three-fourths of cases of this disease, the tumour is clinically confined to the uterus at the time of diagnosis, and most patients with early-staged disease achieve a favourable clinical outcome with surgery alone (Morrow *et al*, 1991; Grigsby *et al*, 1992). However, a significant number of patients with early-staged disease develop localised recurrence or distant metastases, and the patients with recurrence or advanced disease show a poor outcome (Lotocki *et al*, 1983). Several clinicopathological parameters are currently used for the classification of risks for relapse and death, such as surgical stage, histological type, grade, depth of myometrial invasion, cervical stromal invasion, lymph node metastasis, lymph-vascular involvement, and peritoneal cytology (Morrow *et al*, 1991; Grigsby *et al*, 1992). For patients belonging to high or intermediate risk groups defined by these parameters, either postoperative adjuvant radiation therapy, or chemotherapy has been used (Creutzberg *et al*, 2000; Keys *et al*, 2004; Sagae *et al*, 2005). However, selecting the patients that will receive the adjuvant therapy, and its effectiveness, especially its impact on survival remain controversial (Cardenes and Randall, 2003). Thus, in addition to the conventional clinicopathological parameters, the identification of biochemical or molecular markers more strictly related to the intrinsic biological behaviour of endometrial cancer, and the individualisation of adjuvant therapy based on more reliable prognostic indicators, may be helpful in further improving the survival of patients with this disease, as well as in preventing the unnecessary use of adjuvant therapy.

Indoleamine 2,3-dioxygenase (IDO; EC 1.13.11.42) is a haeme-containing intracellular enzyme that catalyses the initial and rate-limiting steps in the metabolism of the essential amino acid tryptophan along the kynurenine pathway (Takikawa *et al*, 1986). Recently, evidence for an immunosuppressive function of IDO has been accumulating. Indoleamine 2,3-dioxygenase is expressed in placental cells during pregnancy and prevents rejection of the allogeneic foetus by depleting tryptophan locally and producing tryptophan metabolites, which causes the apoptosis of maternal alloreactive T cells or natural killer cells that are extremely sensitive to tryptophan shortage (Munn *et al*, 1998). Indoleamine 2,3-dioxygenase is also expressed in certain types of activated macrophages or dendritic cells, and regulates the immune response and induces tolerance (Mellor and Munn, 2004).

In malignancy, Uyttenhove *et al* (2003) demonstrated for the first time that IDO was expressed in various human cancer tissues, and that IDO was involved in protecting tumours from attack by tumour-associated antigen-specific host cytotoxic T cells. Furthermore, it has been reported that IDO was expressed not only by tumour cells themselves, but also by dendritic cells in tumour-draining lymph nodes, and that melanoma patients with IDO-positive cells in lymph nodes had a poor clinical outcome (Munn *et al*, 2004). More recently, it was shown that IDO inhibitors potentiated the antitumour activity of chemotherapeutic agents in mice, suggesting the involvement of IDO in the refractoriness to chemotherapy (Muller *et al*, 2005), consistent with another report showing that IDO was associated with chemoresistance in ovarian cancer (Okamoto *et al*, 2005). Based on the above findings, considerable attention is now being paid to the functional role of IDO in the progression of human cancer and its therapeutic potential as a new prognostic marker or a molecular target. However, there have been few studies on IDO expression in a large number of human tumour samples (Brandacher *et al*, 2006), and its prognostic significance has not yet been well studied.

In the current study, we performed immunohistochemical analysis for IDO expression in a total of 80 tissue samples of endometrial cancer, and demonstrated that high IDO expression was a reliable indicator for disease progression and the poor prognosis of endometrial cancer.

## MATERIALS AND METHODS

### Reagents and antibodies

Mouse monoclonal antibody against human IDO was prepared as described previously (Takikawa *et al*, 1988). L-tryptophan, catalase, ascorbic acid, methylene blue, L-kynurenine, and dimethylaminobenzaldehyde were all purchased from Sigma Chemical Co. (St Louis, MO, USA).

### Immunoblot analysis

Tumour tissue samples were homogenised in a lysis buffer consisting of 1% Triton X-100 and the protease inhibitor mixture in phosphate-buffered saline. After centrifugation at 15 000 *g* for 20 min, the supernatant was obtained. Thirty micrograms of protein extract was separated by SDS/10% polyacrylamide gel electrophoresis, transferred onto the nitrocellulose membrane, and immunoblotted with anti-IDO monoclonal antibody at a dilution of 1 : 1000. Immunoreactive proteins were stained using a chemiluminescence detection system (ECL, Amersham, Arlington Heights, IL, USA).

### Assay for IDO enzymatic activity

IDO enzymatic activity was determined colorimetrically according to the previous reports (Kudo and Boyd, 2000) with minor modifications. Briefly, 0.1 ml of protein extract was mixed with 0.1 ml of the substrate solution composed of 10 mM L-tryptophan, 10 mg ml^−1^ catalase, 200 mM ascorbic acid, 500 *μ*M methylene blue, and 500 mM potassium phosphate buffer (pH 6.5), and incubated at 37°C for 60 min. The reaction was terminated by the addition of 40 *μ*l of 30% trichloroacetic acid and further incubated at 50°C for 15 min to hydrolyse *N*-formylkynurenine produced by IDO to kynurenine. After centrifugation for 20 min at 3000 *g*, 0.1 ml of the supernatant was collected, and then 0.1 ml of 2% dimethylaminobenzaldehyde in acetic acid was added. As the standard assay, 0.1 ml of 100 *μ*M L-kynurenine was mixed with dimethylaminobenzaldehyde. The absorbance at 480 nm for the yellow colour derived from kynurenine was determined.

### Patients and case selection

Eighty patients with endometrial endometrioid adenocarcinoma who underwent surgical treatment at Nagoya University Hospital between 1992 and 2001 were included in this study. Initial diagnoses were made preoperatively by the pathological review of endometrial biopsy or curettage specimens. Surgical treatment consisted of total abdominal hysterectomy and bilateral salpingo-oophorectomy, followed by surgical staging, including peritoneal washing cytology and lymphadenectomy. Patients with the histological cell types other than endometrioid adenocarcinoma, such as papillary serous or clear cell, were not included in this study. The mean age of the patients was 57.2 years (range 31–86). All patients were staged according to the 1988 International Federation of Gynecology and Obstetrics (FIGO) criteria: 54 were stage I (seven were IA, 33 were IB, 14 were IC), 10 were stage II, 10 were stage III and six were stage IV. Histological grade was assigned according to the criteria of the World Health Organization (WHO) classification: 40 were G1 (well differentiated), 27 were G2 (moderately differentiated), and 13 were G3 (poorly differentiated). In this study, all patients with FIGO stage IC and more advanced-staged disease received postoperative adjuvant chemotherapy with six cycles of either cisplatin/doxorubicin/cyclophosphamide or cisplatin plus etoposide in 1992–1999, and carboplatin plus paclitaxel after 2000. Patients receiving postoperative radiation therapy or any preoperative treatment were excluded from this study because the number of these patients was very small. Tumour recurrence/progression was defined based on clinical, radiological or histological diagnosis. Patients with recurrence were treated with the chemotherapy, local radiation therapy, or surgical tumour resection if possible.

### Immunohistochemistry

Informed consent was obtained from individual patients for the use of their tissue samples. Surgical specimens were fixed in 10% formalin and embedded in paraffin. Paraffin specimens were cut at a thickness of 4 *μ*m. For heat-induced epitope retrieval, deparaffinised sections were soaked in Target Retrieval Solution consisting of 10 mM Tris and 1 mM EDTA (DAKO, Glostrup, Denmark), and treated at 95°C for 30 min in a microwave oven. Immunohistochemical staining was performed using the avidin–biotin immunoperoxidase technique. Endogenous peroxidase activity was blocked by incubation with 0.3% H_2_O_2_ in methanol for 15 min, and nonspecific immunoglobulin binding was blocked by incubation with 10% normal goat serum for 10 min. Sections were incubated at room temperature for 2 h with anti-IDO monoclonal antibody at 1 : 200 dilution. The sections were rinsed and incubated for 30 min with the biotinylated second antibody. After washing, the sections were incubated for 30 min with horseradish peroxidase-conjugated streptavidin, and finally treated with 3,3′-diaminobenzidine tetrahydrochloride in 0.01% H_2_O_2_ for 10 min. The slides were counterstained with Meyer’s hematoxylin. As a negative control, the primary antibody was replaced with normal mouse IgG at an appropriate dilution. As a positive control, tissue sections of normal placenta were used as previously reported (Sedlmayr *et al*, 2002).

The IDO expression levels were classified semiquantitatively based on the total scores of the percent positivity of stained tumour cells and the staining intensity. Namely, the percent positivity was scored as ‘0’ if <5% (negative), ‘1’ if 5–30% (sporadic), ‘2’ if 30–70% (focal), and ‘3’ if >70% (diffuse) of cells stained, whereas the staining intensity was scored relative to the known positive and negative controls as ‘0’ if no staining, ‘1’ if weakly stained, ‘2’ if moderately stained (intermediate level between strong and weak), and ‘3’ if strongly stained. The final IDO expression score was defined as follows; ‘IDO−’ if the sum of the percent positivity score and the staining intensity score was 0–1, ‘IDO1+’ if the sum was 2–3, ‘IDO2+’ if the sum was 4–5, and ‘IDO3+’ if the sum was 6. In this scoring system, IDO expression in the tumour stromal cells was not considered because the IDO immunostaining in non-tumour cells was not remarkable or absent, whereas it was dominant in tumour cells, in all cases examined. In each case, at least three different areas were evaluated, and the mean of the results was considered the final expression score. The scoring procedure was carried out twice by two independent observers (each blinded to the other's score) without any knowledge of the clinical data. The concordance rate was over 95% between the observers. In the case of disagreement, the slides were reviewed simultaneously by these two observers, with another, different observer, who were seated together at a multiheaded microscope, in order to resolve the difference of opinion.

### Statistical analysis

Fisher's exact test and Pearson *χ*^2^ test were used to analyse the correlation of IDO expression with various clinicopathological parameters. Overall survival (OS) was calculated from the date of surgery to the date of death, and progression-free survival (PFS) was calculated from the date of surgery to the date of progression/recurrence or date of last follow-up. Survival analyses were performed according to the Kaplan–Meier method. Comparison of the survival between groups was performed with the log-rank test. Cox proportional-hazard analysis was used for univariate and multivariate analysis to explore the effect of variables on survival. The SAS software (SAS Institute Inc., Cary, NC, USA) was used for all statistical analyses, and a *P*-value of <0.05 was considered significant.

## RESULTS

### IDO protein expression and enzyme activity in endometrial cancer tissues

**Figure 1:**
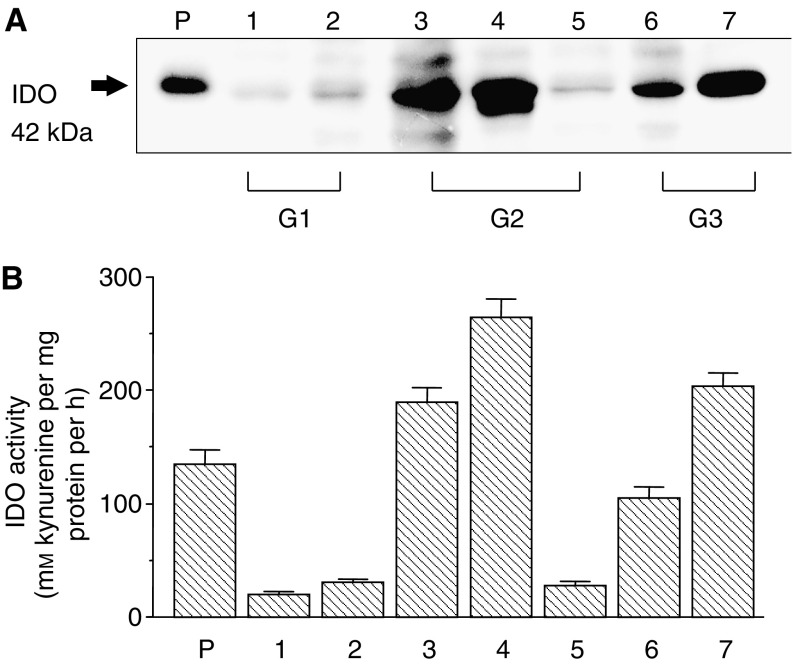
Indoleamine 2,3-dioxygenase expression in endometrial cancer tissues according to Western blot analysis (**A**) and enzyme activity assay (**B**). Lane 1–7 corresponded to the seven different endometrial cancer patients (G1, grade 1; G2, grade 2; and G3, grade 3). P, placental tissue used as a positive control.

First, the IDO protein expression was examined in the endometrial cancer tissues obtained from seven patients using Western blot analysis. In all samples, IDO protein was detected as approximately 42 kDa bands, although its expression level varied among the samples (Figure 1A). To confirm whether IDO expressed in these tissues is enzymatically active, the IDO activity was measured by assessing the degradation of tryptophan to generate kynurenine. The IDO enzyme activity was confirmed in all samples and corresponded to the protein expression level (Figure 1B).

### Immunohistochemical expression of IDO in endometrial cancer tissues

**Figure 2:**
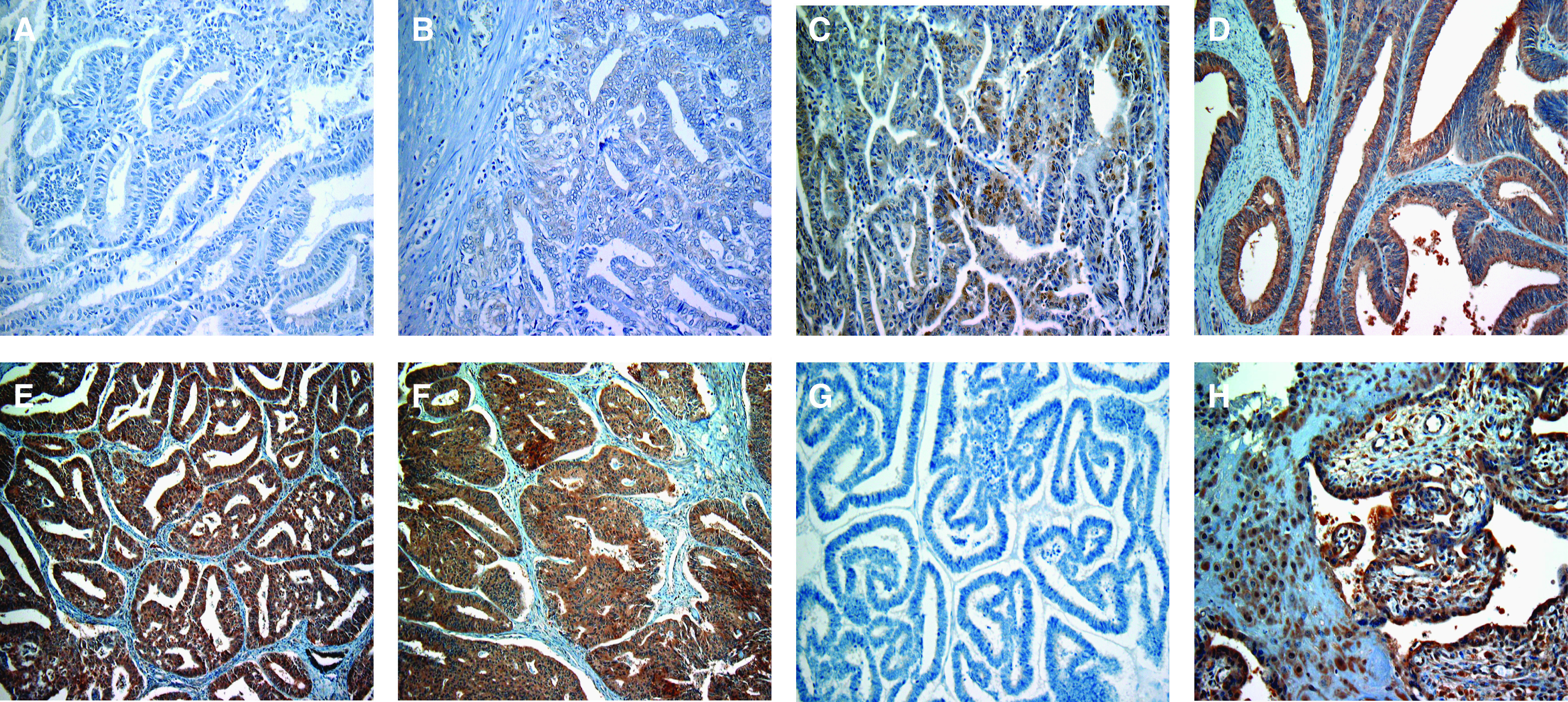
Representative immunohistochemical staining for IDO expression in endometrial cancer tissues. (**A**) IDO− (negative); (**B**) IDO1+ (sporadic/weak); (**C**) IDO2+ (focal/moderate); (**D**–**F**) IDO3+ (diffuse/strong); (**G**) negative control; (**H**) positive control (normal placenta). Original magnification, × 100 in **A**–**H**.

We examined the IDO expression in endometrial cancer by immunohistochemical staining, using a total of 80 surgical specimens. As shown in Figure 2A–F, the immunoreactivity of IDO was detected at variable levels, and was localised in the cytoplasm of tumour cells. In contrast, the immunoreactivity of IDO was very faint or absent in the tumour stroma. Of the 80 specimens examined, the ‘high IDO expression’ (IDO2+ or 3+) were found in 37 (46%) cases, of which 25 (31%) were IDO2+ and 12 (15%) were IDO3+, IDO−, and IDO1+ tumours were found in 15 (19%) and 28 (35%) cases, respectively. The IDO immunoreactivity was not detected in the negative control experiments (Figure 2G), whereas it was strongly detected in the placental tissues used as a positive control (Figure 2H).

The correlation of the high IDO expression (IDO2+ and 3+) with various clinicopathological parameters in the 80 cases are summarised in Table 1. The high IDO expression was positively correlated with the FIGO stage (*P*=0.001), myometrial invasion (*P*=0.001), lymph-vascular space involvement (*P*=0.001) and lymph node metastasis (*P*=0.023), but not with the histological grade.

### Correlation of IDO expression with the patient survival

Follow-up data were available for all 80 patients. The median follow-up period was 71.6 months (range 5–148). During the follow-up period, disease progression/recurrence was observed in 14 cases (17.5%), in which nine patients (11.3%) died. The median time to progression/recurrence and death were 10.5 and 17.8 months, respectively.

**Figure 3:**
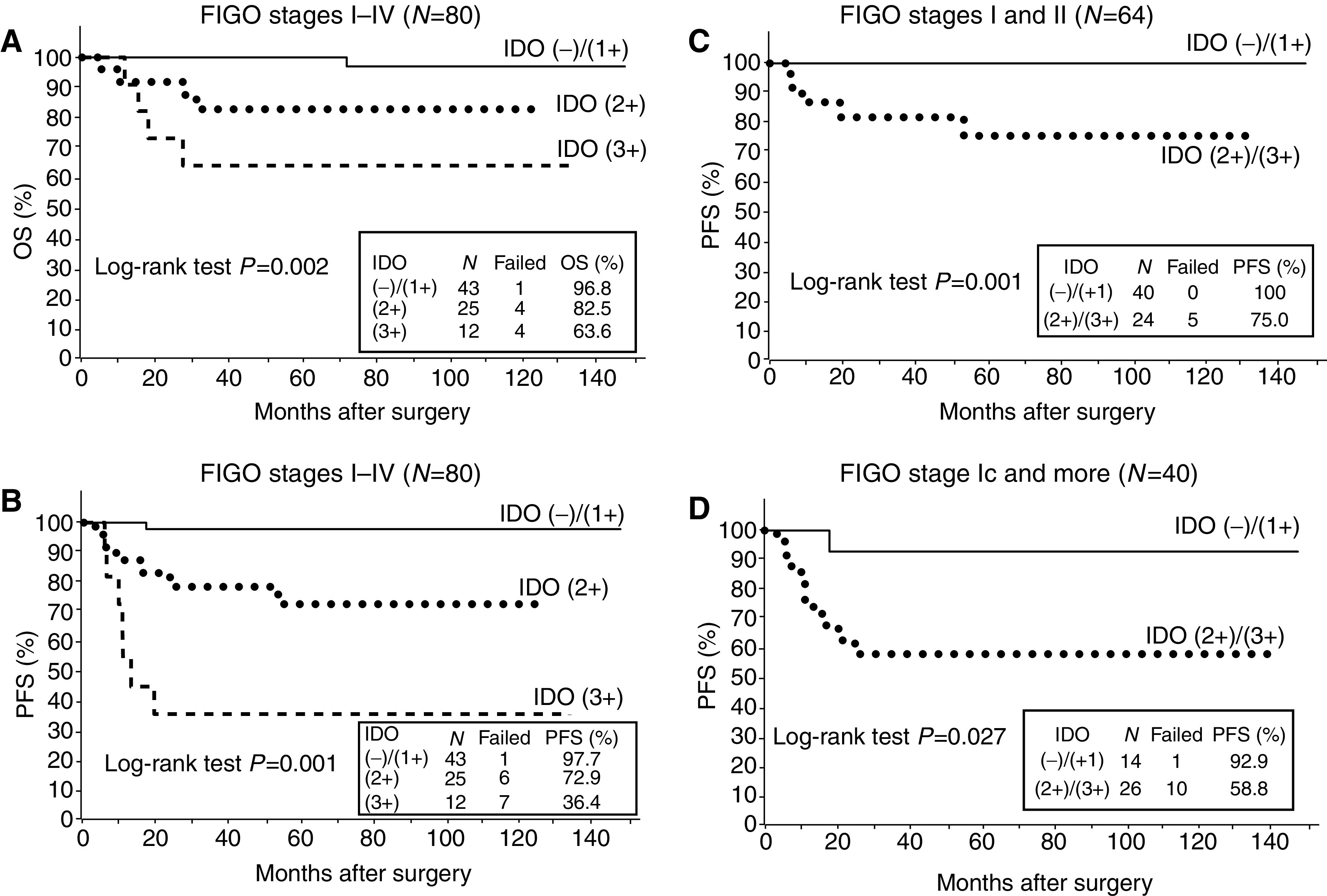
Overall survival and PFS curves drawn using the Kaplan–Meier method according to the IDO expression in endometrial cancer patients. (**A** and **B**) OS and PFS in all patients (*n*=80). Significant differences in the OS (*P*=0.002) and PFS (*P*=0.001) according to the IDO expression among the three groups. (**C**) PFS in stage I–II patients (*n*=64). Significant difference between the two groups (*P*=0.001). (**D**) PFS in stage Ic-IV patients (*n*=40). Significant difference between the two groups (*P*=0.027).

To evaluate the impact of IDO expression on patient prognosis, OS, and PFS curves were constructed using the Kaplan–Meier method. The OS rates of patients with IDO−/1+, IDO2+, and IDO3+ were 96.8, 82.5, and 63.6%, respectively (Figure 3A). The 5-year PFS rates for IDO−/1+, IDO2+, and IDO3+ were 97.7, 72.9, and 36.4%, respectively (Figure 3B). Patients with high IDO expression (IDO2+ or 3+) had significantly impaired OS (*P*=0.002) and PFS (*P*=0.001) as compared to patients with no or weak expression of IDO (IDO− or 1+) (Figure 3A and B).

Next, we analysed the correlation of IDO expression with PFS in the patients with early-staged disease. In FIGO stage I/II patients (*n*=64), the 5-year PFS rates for IDO−/1+ and IDO2+/3+ were 100 and 75.0%, respectively, and there was a significant difference in the PFS between the two groups (*P*=0.001) (Figure 3C). Finally, we analysed the correlation of IDO expression with PFS in the patients with FIGO stage Ic and greater (Ic-IV, *n*=40), because all these patients underwent postoperative adjuvant chemotherapy owing to the risk of disease recurrence/progression. In these patients, the 5-year PFS rates for IDO−/1+ and IDO2+/3+ were 92.9 and 58.8%, respectively, with a marked difference between the two groups (*P*=0.027) (Figure 3D).

### Multivariate analysis of prognostic variables in endometrial cancer patients

Cox proportional-hazard analysis was performed to compare the impact of IDO expression on survival with those of currently used clinicopathological prognostic factors. The results of the univariate/multivariate analyses of the variables, including IDO expression, age, FIGO stage, grade, myometrial invasion, and lymph-vascular space involvement, with respect to OS and PFS are shown in Tables 2 and 3, respectively. Among the six variables, the FIGO stage was the only significant prognostic factor (hazard ratio=5.59, *P*=0.021) with respect to OS on multivariate analysis, although IDO expression, as well as stage, myometrial invasion, and lymph-vascular space involvement, were significant prognostic factors on univariate analysis (Table 2). In contrast, both IDO expression (hazard ratio=12.04, *P*=0.020) and FIGO stage (hazard ratio=4.52, *P*=0.009) were found to be independent prognostic factors with respect to PFS on multivariate analysis (Table 3).

## DISCUSSION

In the present study, we demonstrated the expression of the tryptophan-catabolising and immunosuppressive enzyme, IDO, in endometrial cancer using 80 surgical specimens, and found that the high IDO expression by tumour cells was positively correlated with disease progression and the impaired patient survival. This is the first study to demonstrate the detailed clinicopathological and prognostic impact of IDO expression in human cancer using a large number of clinical samples.

Prior studies showed that IDO was barely expressed in the proliferative phase of the normal endometrium, whereas its expression was increased in the secretory phase, suggesting that IDO expression in the normal endometrium is physiologically regulated, dependent on the menstrual cycle (Sedlmayr *et al*, 2002; Kudo *et al*, 2004). In contrast, the present study showed that the frequency of IDO expression in endometrial cancer was high. Indeed, IDO was highly expressed in 37 out of 80 (46%) cases, whereas IDO-negative tumours were found in only 15 (19%) cases. Several studies showed that IDO was localised in tumour cells, as well as in some macrophages and eosinophil granulocytes in the tumour stroma, or in dendritic cells in tumour-draining lymph nodes (Friberg *et al*, 2002; Munn *et al*, 2004; Astigiano *et al*, 2005). In our study, the localisation of IDO was dominant in tumour cells, and the IDO expression in the tumour stroma was not prominent or was absent. Consistently, Uyttenhove *et al* (2003) showed that IDO was expressed in the cancer cell itself, in a variety of human tumour types. In gynaecological malignancies, IDO expression was detected in cervical, endometrial, and ovarian cancers with a high rate of positivity, although a very limited number of samples were analysed in prior studies (Sedlmayr *et al*, 2003; Uyttenhove *et al*, 2003). Recently, Okamoto *et al* (2005) reported that IDO was expressed in 17 out of 24 cases with advanced ovarian carcinoma. Schroecksnadel *et al* (2005) also showed that the serum tryptophan concentration was lower in 20 patients with gynaecological cancer, suggesting the presence of tumour-mediated IDO activity in these patients. Taken together with our results, IDO is frequently expressed in gynaecological cancers.

We analysed the correlation of IDO expression with the clinicopathological parameters in endometrial cancer. The high IDO expression was significantly correlated with the advanced stage, the depth of myometrial invasion, the presence of lymph-vascular space involvement, and lymph node metastasis, but not with the histological grade. Consistently, Brandacher *et al* (2006) showed that the high IDO expression in colorectal cancer was correlated with the disease stage and liver metastasis, but not with tumour differentiation. These results suggest that the high IDO expression is associated with the disease progression of endometrial cancer, and it might reflect the local tumour aggressiveness rather than the grade of tumour differentiation.

The present data clearly demonstrated that the patients with high IDO expression had an impaired clinical outcome by analysing the rates of both OS and PFS. Surprisingly, of the 43 patients with no or weak IDO expression, only one patient developed disease progression/recurrence, whereas 13 out of the 37 patients with high IDO expression recurred. Consistently, three recent reports showed the correlation between the high IDO expression and poor patient prognosis in ovarian cancer, lung cancer, and colorectal cancer (Astigiano *et al*, 2005; Okamoto *et al*, 2005; Brandacher *et al*, 2006), although they only analysed the OS. More importantly, our analysis, focusing on the cases with early-staged disease (FIGO I/II), showed that the patients with no or weak expression of IDO achieved 100% PFS, whereas five out of 24 patients with the high IDO expression developed recurrence (PFS=75%), and three out of the five recurred cases were FIGO stage 1B. These findings suggested that the patients with high IDO expression are very likely to recur and have poor prognosis, even in cases with early-staged disease at the time of surgery. Furthermore, our multivariate analysis demonstrated that only IDO expression, except for the stage, was an independent prognostic factor for PFS, suggesting that IDO may be a more reliable prognostic parameter of endometrial cancer than the currently used clinicopathological factors. Because over 70% of endometrial cancer patients present early-staged disease, and most of them are curable with surgery alone, it would be of substantial benefit to define the minority of patients who are likely to recur, and also to give aggressive adjuvant therapy to these patients alone. There have been several molecular markers identified showing the prognostic impact in endometrial cancer (Mell *et al*, 2004; Shibata *et al*, 2004; Stefansson *et al*, 2004; Ferguson *et al*, 2005); however, their clinical application instead of, or in addition to, the currently used prognostic factors has not yet been realised. Our data suggests that IDO may become a useful indicator for the prognosis of endometrial cancer and may contribute to the individualisation of the application of adjuvant therapy, not only in advanced staged, but also in early staged patients. Furthermore, it might contribute to the cost–benefit by preventing the unnecessary use of adjuvant therapy.

The mechanism by which IDO expression contributes to the tumour progression of endometrial cancer remains to be determined. Recent reports proposed a mechanism that IDO can induce the growth inhibition of tumour antigen-specific cytotoxic T cells by depleting the tumour microenvironment of tryptophan and/or by generating toxic tryptophan metabolites, which causes the tumour to escape from the host immune systems, leading to the uncontrolled tumour progression (Uyttenhove *et al*, 2003; Munn *et al*, 2004). Thus, the correlation of the high IDO expression with disease progression and the impaired patient survival shown by the present study may be attributable to the immune suppression by the tumour-mediated IDO activity. This may be supported by one recent report showing the association of high IDO expression with a reduction of CD3+ lymphocytes in colorectal cancer (Brandacher *et al*, 2006). Another possible mechanism by which IDO contributes to cancer progression was proposed by Muller *et al* (2005), who demonstrated the involvement of IDO in a chemoresistance in cancer. Consistently, Okamoto *et al* (2005) showed that IDO was overexpressed in paclitaxel-resistant ovarian cancer cell lines and tissues using a gene expression profiling study. Our results demonstrated that there was a marked difference (92.9 *vs* 58.8%) in PFS between the no or low IDO expression group and the high IDO expression group when focusing on the 40 patients with FIGO stage Ic and more who underwent postoperative chemotherapy. This might be due, at least in part, to the resistance of IDO-overexpressing endometrial cancer cells to chemotherapy. However, to identify the functional roles of IDO in cancer cells besides its enzymatic or immunosuppressive actions, further studies are needed.

In conclusion, we demonstrated here that high IDO expression correlated with the disease progression and impaired clinical outcome in endometrial cancer patients. Furthermore, IDO was an independent prognostic factor for PFS. These results indicate that IDO is a reliable and promising prognostic indicator and may become a novel molecular target in the strategy for the treatment of endometrial cancer.

## Figures and Tables

**Table 1:** Correlation of IDO expression with clinicopathological factors in endometrial cancer

	**Patients**		**High IDO expression**		
**Characteristics**	**No.**	**%**	**No.**	**%**	***P*-value**
All cases	80	100	37	46.3	
					
*Age (years)*					
>60	51	63.8	23	45.1	0.819
⩾60	29	36.3	14	48.3	
					
*FIGO stage*					
I–II	64	80.0	24	37.5	0.001
III–IV	16	20.0	13	81.3	
					
*Histological grade*					
G1	40	50.0	13	32.5	
G2	27	33.8	17	63.0	0.118
G3	13	16.3	7	53.8	
					
*Myometrial invasion*					
None	7	8.8	1	14.3	
Inner half	44	55.0	15	34.1	0.001
Outer half	29	36.3	21	72.4	
					
*Lymph-vascular space involvement*					
Yes	30	37.5	23	76.7	0.001
No	50	62.5	14	28.0	
					
*Lymph node metastasis*					
Yes	6	7.5	6	100.0	0.023
No	74	92.5	31	41.9	

FIGO=International Federation of Gynecology and Obstetrics; IDO=indoleamine 2,3-dioxygenase.

**Table 2:** Univariate and multivariate analyses of OS in endometrial cancer patients

		**Univariate analysis**			**Multivariate analysis**		
**Variables**	**Categories**	**HR^a^**	**95% CI^b^**	***P*-value**	**HR^a^**	**95% CI^b^**	***P*-value**
Age	>60 years	0.24	0.03–1.88	0.173	—	—	—
	⩾60 years						
							
FIGO stage	I/II	10.51	2.61–42.30	0.001	5.59	1.30–23.99	0.021
	III/IV						
							
IDO expression	0/1+	12.47	1.55–100.49	0.018	6.64	0.75–59.14	0.089
	2+/3+						
							
Histological grade	1 or 2	2.89	0.72–11.55	0.134	—	—	—
	3						
							
Myometrial invasion	None/inner half	4.09	1.02–16.37	0.047	—	—	—
	Outer half						
							
Lymph-vascular space involvement	Yes	6.86	1.42–33.08	0.016	—	—	—
	No						

OS=overall survival.

a HR=hazard ratio.

b CI=confidence interval

**Table 3:** Univariate and multivariate analyses of PFS in endometrial cancer patients

		**Univariate analysis**			**Multivariate analysis**		
**Variables**	**Categories**	**HR**	**95% CI**	***P*-value**	**HR**	**95% CI**	***P*-value**
Age	>60 years	0.30	0.07–1.32	0.110	—	—	—
	⩾60 years						
							
FIGO stage	I/II	9.33	3.11–28.01	0.001	4.52	1.46–13.96	0.009
	III/IV						
							
IDO expression	0/1+	20.51	2.68–157.13	0.004	12.04	1.48–97.79	0.020
	2+/3+						
							
Histological grade	1 or 2	1.58	0.44–5.66	0.484	—	—	—
	3						
							
Myometrial invasion	None/inner half	2.68	0.93–7.75	0.068	—	—	—
	Outer half						
							
Lymph-vascular space involvement	Yes	3.55	1.19–10.60	0.023	—	—	—
	No						

HR=hazard ratio; CI=confidence interval; PFS=progression-free survival.
